# Hospital malnutrition at pediatric ward Dr. Wahidin Sudirohusodo Hospital Makassar

**DOI:** 10.1186/1687-9856-2013-S1-P49

**Published:** 2013-10-03

**Authors:** Aidah Juliaty Wahyudin, JS Lisal

**Affiliations:** 1 Department of Child Health, Faculty of Medicine, Hasanuddin University, Dr. Wahidin Sudirohusodo Hospital, Makassar, Indonesia

## Background

Hospital Malnutrition (HM) is one of the health problems in hospital care. Malnutrition in hospital leads to many problems like prolonged length of stay, clinical complications, delayed recovery and increase economic cost.

## Objective

To determine nutritional status on admission and monitoring their weight changes, in order to reveal incidence of hospital malnutrition.

## Methods

A prospective study was conducted in January until December 2011 period. The initial measurement were made by residents in charge. The nutritional status was defined according to CDC 2000 guidelines. Exclusion criteria including children with fluid retention, organomegaly, obesity, and tumours. HM was defined as weight decreased ≥ 2% for LOS (length of stay) less than 7 days, ≥ 5% for LOS 7 to 30 days and ≥ 10% for LOS 1 to 6 months. Data were analyzed using SPSS for Windows.

## Results

From 1286 patients hospitalized during the period, most of them (81,5%) suffer for weight loss ≥ 2%. According to age, HM occurs more in 25 – 36 months age group (0,16%).

## Conclusion

Incidence of HM still high, a close monitoring to patients intake and needs is necessary to prevent and to lower the incidence.

